# CK2-Mediated Phosphorylation Upregulates the Stability of USP13 and Promotes Ovarian Cancer Cell Proliferation

**DOI:** 10.3390/cancers15010200

**Published:** 2022-12-29

**Authors:** Juntae Kwon, Jinmin Zhang, Boram Mok, Cecil Han

**Affiliations:** 1 Department of Oncology, Georgetown University School of Medicine, Washington, DC 20007, USA; 2 Lombardi Comprehensive Cancer Center, Georgetown University School of Medicine, Washington, DC 20007, USA

**Keywords:** ovarian cancer, USP13, post-translational modification, phosphorylation

## Abstract

**Simple Summary:**

Ubiquitin-specific Peptidase 13 (USP13) is highly amplified and promotes tumorigenesis and metastasis in ovarian cancer. However, post-translational modifications and their functions on USP13 are largely unknown. This study revealed that USP13 is phosphorylated at Thr122, which upregulates the stability of USP13 at a post-translation level. Notably, mutation of Thr122 diminished the effect of USP13 on the increased proliferation of ovarian cancer cells. Overall, we uncovered a new mechanism for the regulation of USP13 stability via a post-translational modification, suggesting novel therapeutics targeting USP13 in *USP13*-amplified cancers.

**Abstract:**

Ubiquitin-specific Peptidase 13 (USP13) is a deubiquitinating enzyme that regulates the stability or function of its substrate. USP13 is highly amplified in human ovarian cancer, and elevated expression of USP13 promotes tumorigenesis and metastasis of ovarian cancer. However, there is little known about USP13 post-translational modifications and their role in ovarian cancer. Here, we found that USP13 is phosphorylated at Thr122 in ovarian cancer cells. Phosphorylated Thr122 (pT122) on endogenous USP13 was observed in most human ovarian cancer cells, and the abundance of this phosphorylation was correlated to the total level of USP13. We further demonstrated that Casein kinase 2 (CK2) directly interacts with and phosphorylates USP13 at Thr122, which promotes the stability of USP13 protein. Finally, we showed that Threonine 122 is important for cell proliferation of ovarian cancer cells. Our findings may reveal a novel regulatory mechanism for USP13, which may lead to novel therapeutic targeting of USP13 in ovarian cancer.

## 1. Introduction

High-Grade Serous Ovarian Cancer (HGSOC) is the most commonly diagnosed form of ovarian cancer and the leading cause of gynecological cancer deaths [1,2]. Not much improvement has been achieved in overall survival for patients with HGSOC in the past thirty years, and standard therapy has not advanced beyond platinum-based combination chemotherapy [3,4]. Therefore, there are urgent challenges in understanding ovarian cancer initiation and progression and finding new ways to target ovarian cancer. 

As a reversible ubiquitination process, deubiquitinating enzymes (DUBs) remove ubiquitin from ubiquitin-modified protein substrates and impact their function, localization, or stability [5,6,7,8]. A deubiquitinating enzyme, Ubiquitin-specific Peptidase 13 (USP13), is known to be engaged in diverse cellular processes by regulating various important substrates implicated in disease and cancer [9,10,11,12,13,14,15,16,17,18,19,20,21,22,23]. USP13 gene copy is frequently amplified in several human cancers, such as lung, esophagus, and ovarian cancer [12,18,24]. Among 50 different ubiquitin-specific peptidases (USPs), USP13 gene copy is strongly amplified (amplification 26% and gain 49%) in HGSOC [12]. High expression of USP13 protein is positively correlated with poor survival outcomes in ovarian cancer patients [12]. We previously demonstrated that USP13-amplified ovarian cancer cells rely on the USP13-associated deubiquitination pathway for their proliferation and survival [12]. In our genetically engineered mouse model, USP13 overexpression promoted ovarian tumorigenesis and metastasis [14]. Post-translational modifications (PTMs) on DUBs affect the function by altering their localization, enzyme activity, or interactome [25,26,27]. Although the diverse roles of USP13 have been studied in various cancer types, including ovarian cancer, the regulatory mechanism of PTMs on USP13 is little known.

Casein kinase 2 (CK2) phosphorylates hundreds of proteins in various subcellular compartments [28,29]. CK2 is involved in various crucial cell events, including gene transcription, signal transduction, cytoskeletal structure regulation, and cell adhesion [30]. CK2 is known to be a constitutively active kinase that is expressed ubiquitously in eukaryotes, while CK2 is abnormally elevated in many cancers [31]. CK2 has been raised as an attractive therapeutic target for treating solid tumors and hematologic malignancies [32]. However, little is known about its role in the ubiquitin–proteasome system, and no link between CK2 and USPs has been elucidated.

Here, we report that USP13 is a CK2 substrate and that this phosphorylation stabilizes USP13 protein both in vitro and in ovarian cancer cells. We also reveal that phosphorylation at Threonine 122 of USP13 promotes the proliferation of ovarian cancer cells. Our study proposes that targeting phosphorylation could inhibit the oncogenic function of USP13 in ovarian cancer. 

## 2. Results

### 2.1. Identification of USP13 Phosphorylation in Ovarian Cancer Cells by LC-MS-MS Analysis

**Figure 1:**
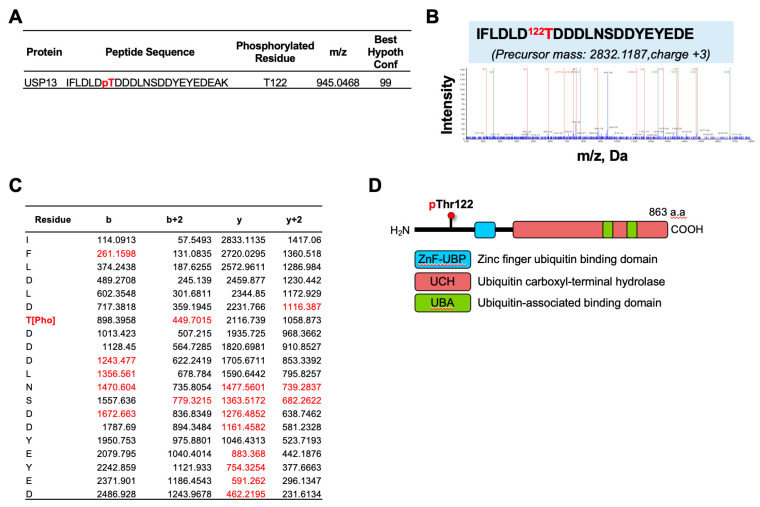
Identification of new phosphorylation site on USP13. (**A**) USP13 phosphorylation site identified by LC-MS/MS in HeyA8 ovarian cancer cells. Peptide sequence, phosphorylation residue, mass-to-charge ratio (*m*/*z*), and best hypothesis configuration are indicated. (**B**) MS/MS spectrum of IFLDLDTDDDLNSDDYEYEDE peptide with phosphorylation at Thr122 with ions (b = green, y = red). Both b-type and y-type ions were labeled to determine the peptide sequence of the precursor ion and to locate the sites of phosphorylation. (**C**) Matched b-type and y-type ions are shown in red, with phosphorylation at Thr122 unambiguously assigned. b-ions: the charge is retained by the amino-terminal part of the peptide. y-ions: the charge is retained by the carboxyl-terminal part of the peptide. (**D**) USP13 domain structure and Threonine 122 (Thr122) phosphorylation site. Thr122 is located at the N-terminal of USP13 protein, but not in the conserved functional domains.

To identify phosphorylation sites on USP13 in ovarian cancer, we performed immunoprecipitation (IP) of endogenous USP13 in human ovarian cancer HeyA8 cells. USP13 IP samples were run in SDS-PAGE gel, and in-gel digestion was performed. The digest was enriched for phosphopeptides and further analyzed with tandem mass spectrometry. The mass spectrometry identified a phosphorylated signal at the Threonine 122 residue (Thr122) of USP13 with confidence >95% (Figure 1A). A phosphopeptide IFLDLD122TDDDLNSDDYEYEDEAK (observed m/z 2832.1187) was identified using Protein Pilot version 5.0 software (Sciex) (Figure 1B). The matched fragment ions (red in Figure 1C) indicated that phosphorylation was on Thr122. Threonine 122 is located at the N-terminal of USP13 (Figure 1D). These data indicate that the phosphorylation occurs to the Thr122 residue of USP13 in HeyA8 ovarian cancer cells.

### 2.2. Generation of a Thr122 Phospho-Specific USP13 Antibody

To study the function of phosphorylation on Thr122 of USP13 in ovarian cancer, we generated an anti-phospho-Thr122 USP13 antibody (anti-pT122 antibody). The synthesized phosphopeptide was used to immunize rabbits (CRRNSKIFLDLD(pThr)DD). Anti-pT122 antibody was purified by using both phosphopeptide and non-phosphopeptide (CRRNSKIFLDLDTDD).

**Figure 2:**
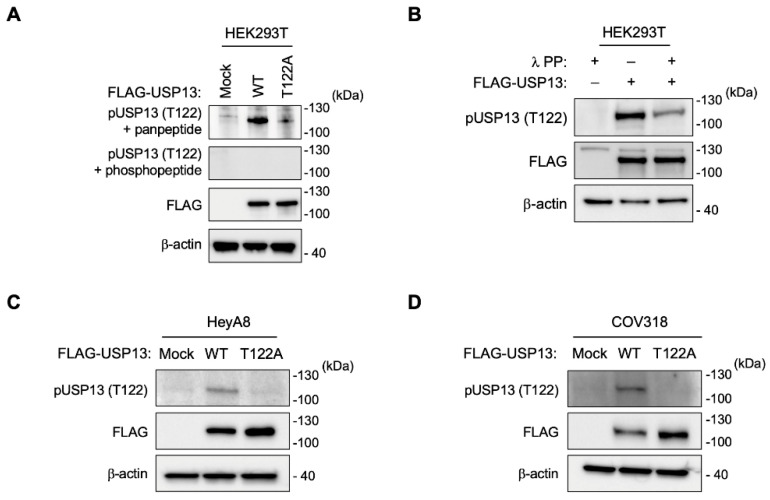
USP13 is phosphorylated at Thr122 in human HEK293T and ovarian cancer cells. (**A**) Analysis of anti-pT122 antibody specificity using peptide competition assay on western blotting (WB) (**B**) WB analysis of HEK293T cells transfected with FLAG-USP13 and preincubated with or without λ-phosphatase as indicated. FLAG-tagged wild-type USP13 (WT) or FLAG-tagged phospho-mutant USP13 (T122A) was transfected into HeyA8 (**C**) and COV318 (**D**) ovarian cancer cells. WB measured the levels of pT122 and FLAG-USP13. β-Actin was used as a loading control.

To validate the activity of the anti-pT122 antibody, we generated the point mutation of Thr122 by using a site-directed substitution of threonine to alanine on USP13 to abolish USP13 phosphorylation on Thr122 residue. The anti-pT122 antibody detected a specific phosphorylated signal of USP13 in HEK293T cells expressing USP13 wild-type but not in HEK293T cells expressing phospho-mutant (T122A) USP13 (Figure 2A). Peptide competition assay further validated the specificity of this antibody against USP13 phosphorylation at Thr122 (Figure 2A). The reactivity of USP13 with anti-pT122 antibody was significantly reduced after incubation with lambda phosphatase (Figure 2B).

### 2.3. Phosphorylation of USP13 on Thr122 in Ovarian Cancer Cells

**Figure 3:**
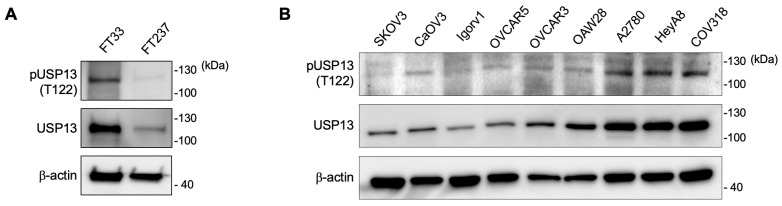
Endogenous USP13 is phosphorylated at Thr122 in various human ovarian cancer cells. Total USP13 and pUSP13 (T122) were detected by western blotting. (**A**) USP13 was phosphorylated at T122 in human fallopian tube secretory epithelial cells. (**B**) Protein lysates were prepared in 9 human ovarian cancer cells. pUSP13 (T122) was detected in all ovarian cancer cells, and the phosphorylation level at Thr122 increased as the total USP13.

We overexpressed FLAG-tagged wild-type USP13 (WT) and phospho-mutant USP13 (T122A) in two human ovarian cancer cell lines. The anti-pT122 antibody specifically detected a phosphorylated USP13 in HeyA8 and COV318 expressing wild-type USP13, not T122A mutant USP13 (Figure 2C,D). Next, we examined the phosphorylated Thr122 (pT122) on endogenous USP13 in immortalized human fallopian tube secretory epithelial cells and various human ovarian cancer cell lines with the validated anti-pT122 antibody. We observed that USP13 is phosphorylated at Thr122 residue in the human fallopian tube epithelial cells (Figure 3A). Thr122 phosphorylation was observed in most ovarian cancer cell lines (Figure 3B). The pT122 level was variable across different ovarian cancer cell lines. The abundance of the phosphorylated form (pT122) of USP13 was correlated to the total level of USP13 in most ovarian cancer cells.

### 2.4. Casein Kinase 2 Is Responsible for Thr122 Phosphorylation on USP13

**Figure 4:**
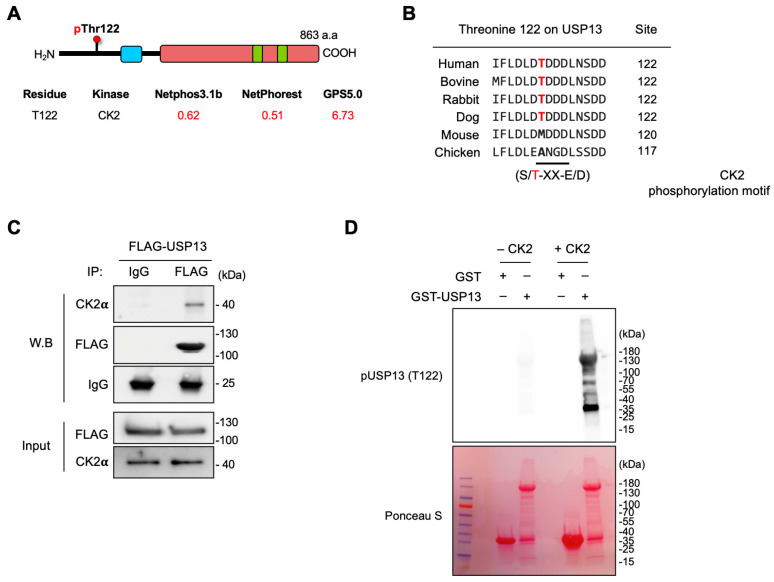
CK2 interacts and phosphorylates USP13 at Thr122 in vitro. (**A**) Prediction of putative kinase against Thr122 was performed by using NetPhos server 3.1, NetPhorest, and GPS5.0. Three software predicted CK2 as a kinase for Thr122 with a high score (red). (**B**) Alignment of USP13 homologous sequences showing the CK2 phosphorylation consensus site. The amino acid at the location corresponding to Thr122 of human USP13 is marked in bold. Amino acid numbering is according to the USP13 sequence. (**C**) USP13 interacts with CK2α. The protein–protein interaction of USP13 and CK2α was confirmed by immunoprecipitation. (**D**) CK2 phosphorylates USP13 in vitro. Bacterially expressed and purified USP13 proteins were incubated with active CK2α in the presence of ATP. Reaction products were separated by SPS-PAGE, and an anti-pT122 antibody was used to detect phosphorylated USP13. The amount of GST and GST-USP13 protein was shown by Ponceau S red staining.

Casein kinase 2 (CK2), a highly conserved serine/threonine kinase, is a tetrameric complex consisting of two catalytic (α and α′) subunits and two regulatory (β) subunits [32]. CK2 phosphorylates serine or threonine residues within an acidic context [30]. In silico analyses identified that Thr122 is located in a phosphorylation consensus site (S/TXXE/D) of CK2, predicting CK2 as a specific kinase for this residue (Figure 4A). The Thr122 phosphorylation site was conserved across different mammalian species (Figure 4B). To determine whether USP13 interacts with CK2 in ovarian cancer cells, we examined CK2–USP13 binding in HeyA8 cells expressing FLAG-tagged USP13. USP13 was pulled down by anti-FLAG antibody in HeyA8 cells and then analyzed with western blotting with anti-CK2α antibody. This experiment showed that CK2 was co-immunoprecipitated with USP13, indicating that USP13 interacts with CK2 in ovarian cancer cells (Figure 4C). We performed in vitro kinase assay using purified recombinant USP13 expressed in *Escherichia. Coli* (*E. coli*) and enzymatically activated CK2 holoenzyme. The recombinant GST or GST-USP13 full-length was expressed from *E. coli* BL21 and immobilized on glutathione-Sepharose beads. In vitro kinase assays were performed directly on the GST or GST-USP13 beads with active CK2 enzyme and ATP. Western blotting with an anti-pT122 antibody showed that CK2 phosphorylated GST-USP13 protein but not GST, indicating that USP13 is a substrate of CK2 in vitro (Figure 4D), suggesting that USP13 is a substrate for CK2.

### 2.5. Inhibition of CK2 Decreased Thr122 Phosphorylation on USP13

**Figure 5:**
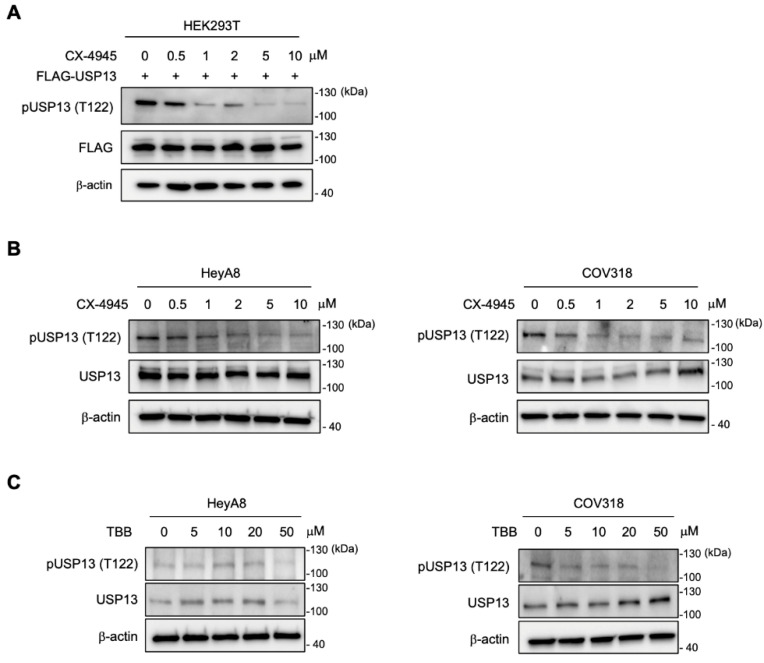
Inhibition of CK2 decreased USP13 phosphorylation at Thr122. (**A**) Immunoblot analysis of HEK293T cells expressing FLAG-USP13 after treatment of CX-4945 for 24 h. The treatment of CX-4945 dramatically reduced the level of Thr122 phosphorylation. β-actin was included as a loading control. (**B**) Treatment of CX-4945 (for 24 h) and (**C**) TBB (for 48 h) reduced phosphorylation of endogenous USP13 at Thr122 in two different human ovarian cancer cells; HeyA8 (left) and COV318 (right).

We then addressed whether CK2 phosphorylates USP13 in the cellular context by inhibiting CK2 kinase activity in cells with CK2 inhibitors, silmitasertib (CX-4945) and 4,5,6,7-tetrabromobenzotriazole (TBB) [33,34,35]. We first determined an effective dose of the inhibitor by treating HEK293T cells expressing FLAG-tagged USP13 with increasing concentration of CX-4945. Cell lysates were prepared the following day and immunoblotted with an anti-pT122 antibody. The treatment with CK2 inhibitor (CX-4945) dramatically reduced the signal of Thr122 phosphorylation of USP13 in HEK293T cells (Figure 5A). The result indicated significant inhibition of USP13 phosphorylation on Thr122 at a concentration from 1 µM of CX-4945. Next, two ovarian cancer cells (HeyA8 and COV318) were treated with CX-4945 for 24 h and TBB for 48 h. Both CK2 inhibitors successfully decreased the abundance of Thr122 phosphorylation in ovarian cancer cells (Figure 5B,C). CX-4945 (0.5–1 µM) more effectively decreased Thr122 phosphorylation in both cell lines, while TBB was effective at 5–50 µM concentrations.

### 2.6. Thr122 Phosphorylation Upregulated USP13 Stability

**Figure 6:**
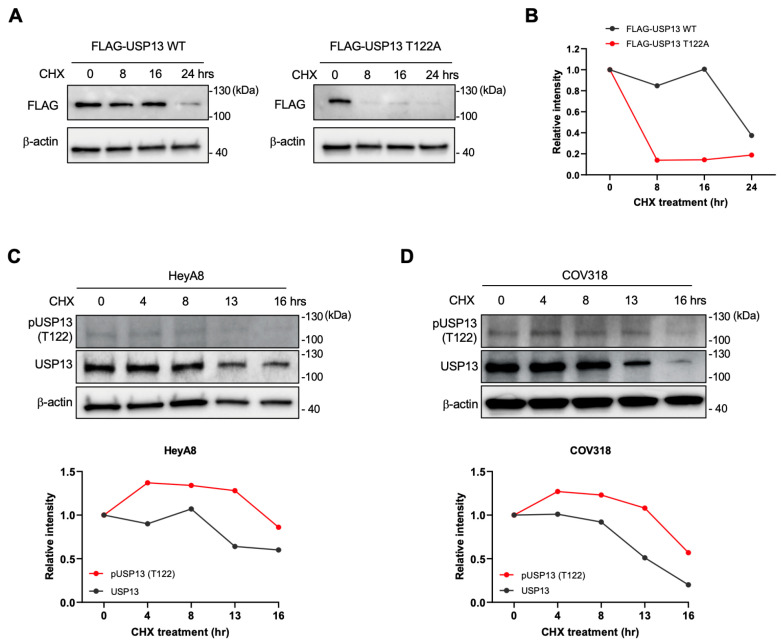
CK2-mediated phosphorylation affects USP13 stability. (**A**) Immunoblot analysis of HEK293T cells transfected with wild-type USP13 (left) or phospho-mutant USP13 (T122A) (right) followed by CHX (100 µg/mL) treatment for indicated times. (**B**) Quantification of the band intensities in the (A). Band intensity was normalized to the β-actin. (**C**,**D**) The stability of total USP13 and pT122 USP13 was measured in ovarian cancer cells. Hey8A (**C**) or COV318 (**D**) were treated with CHX (100 µg/mL) for indicated times. The protein level was examined by western blotting (top) and quantified in the graph (bottom).

The phosphorylation at Thr122 of USP13 could result in various effects on USP13. We examined if the phosphorylation of Thr122 affects the stability of USP13. HEK293T cells were transfected with FLAG-tagged wild-type USP13 (WT) or FLAG-tagged phospho-mutant USP13 (T122A) and then treated with 100 µg/mL of cycloheximide (CHX). In CHX chase assays, the exogenously expressed wild-type of USP13 was stable over 16 h. However, the phospho-mutant of USP13 (T122A) showed a significantly down-regulated half-life time in HEK293T cells (Figure 6A,B). Next, we measured the endogenous levels of total USP13 and phosphorylated USP13 (pT122) in HeyA8 and COV318 cells in the presence of 100 µg/mL CHX. CHX chase assay revealed that the phosphorylated USP13 at Thr122 was more stable than the total USP13 in ovarian cancer cells (Figure 6C,D). Altogether, these data suggest that phosphorylation at the T122 residue of USP13 increases the protein stability of USP13.

### 2.7. Thr122 Phosphorylation Is Important for the Proliferation of Ovarian Cancer Cells

**Figure 7:**
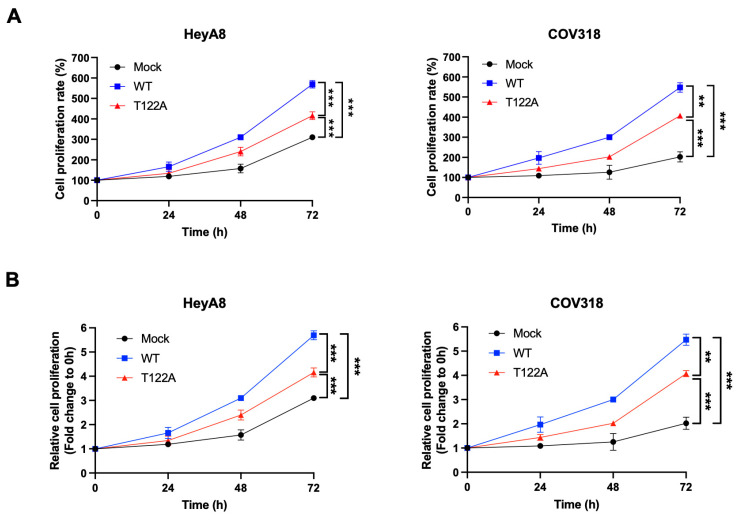
Thr122 phosphorylation of USP13 promotes the proliferation of ovarian cancer cells HeyA8 and COV318 cells were transfected with wild-type USP13 (WT) and phospho-mutant USP13 (T122A). Overexpression of both wild-type USP13 and phospho-mutant USP13 (T122A) in HeyA8 and COV318 cancer cells significantly increased cell proliferation. T122A mutant showed a reduced proliferation rate compared with USP13 wild-type. The cell proliferation at different time points was examined using cell number counting (**A**) and MTT assay (**B**). Statistical data are presented as mean ± SD. Two-tail unpaired Student’s *t*-test was conducted for statistical analysis (n = 3) (** *p*-value < 0.01, *** *p*-value < 0.001).

To understand the biological role of Thr122 phosphorylation of USP13 in ovarian cancer, we examined the effect of inhibiting T122 phosphorylation on cell proliferation. The wild-type USP13 and phospho-mutant USP13 (T122A) were overexpressed in HeyA8 and COV318 cells, and the cell numbers were counted at different time points to determine cell proliferation. The wild-type USP13 and phospho-mutant T122A increased cell proliferation compared to the mock sample in HeyA8 and COV318 cell lines (Figure 7A,B). However, the mutation of Thr122 significantly reduced the effect of USP13 (WT) on the increased cell proliferation. These data suggests that Thr122 phosphorylation is important for USP13 oncogenic function in ovarian cancer cells.

## 3. Discussion

We report here a new manner of USP13 regulation involving its phosphorylation by protein kinase CK2. CK2 phosphorylates the Thr122 residue of USP13, which upregulates the stability of USP13 at a post-translation level. Moreover, this phosphorylation promoted the proliferation of ovarian cancer cells. Our data suggest that inhibiting Thr122 phosphorylation of USP13 may provide an effective therapeutic strategy for targeting USP13 in USP13-amplified ovarian cancer.

Although the experimental results showed that USP13 could bind ubiquitin, bacterial-purified recombinant USP13 still exhibited only weak deubiquitination enzyme activity [16,36,37]. This is incompatible with the findings that USP13 can deubiquitinate diverse substrates implicated in human disease and various cancer [36]. These suggest that post-translational modifications (PTMs) of USP13 may regulate directly deubiquitinating enzyme activity or the interaction with its substrates for efficient function. Indeed, previous reports have shown that PTMs are critical for USP13 activity and function in eukaryote cells. USP13 is phosphorylated at Threonine 196 by ATM in response to the DNA damage response, which facilitates its recruitment to DNA double-strand breaks in HEK293T cells [15]. The phosphorylation of USP13 at Tyrosine 708 by CLK3 is required for USP13′s binding to c-Myc, which prevents its Fbxl14-mediated ubiquitination of c-Myc in glioma stem cells [10]. CLK3-mediated phosphorylation of USP13 at Tyr708 promotes cholangiocarcinoma progression by activating c-Myc-induced purine synthesis, providing a new and viable therapeutic target for treating cholangiocarcinoma associated with CLK3 mutations [33]. Aurora B phosphorylates USP13 at Serine 114, prompting their interaction between USP13 and Aurora, showing a potential direct link between abnormalities occurring during the cell cycle and USP13 in several human cancers [34].

Currently, little is known about the molecular mechanism regulating USP13 protein stability. Our data demonstrated that CK2-mediated phosphorylation at Thr122 regulates the half-life of the USP13 protein in ovarian cancer cells. CHX chase assay data suggest that Thr122 phosphorylation upregulates the USP13 stability at the post-translation level (Figure 6). Thr122 phosphorylation could affect the ubiquitination status of USP13 by inhibiting the binding of E3 ubiquitin ligase or promoting the binding of deubiquitinase on USP13. USP13 may also directly act on removing the ubiquitin level with its own deubiquitinase activity. The mutation of the Thr122 phosphorylation site of USP13 reduced the proliferation of ovarian cancer cells compared with USP13 wild-type, suggesting that Thr122 phosphorylation is important for the oncogenic function of USP13 in ovarian cancer (Figure 7). Furthermore, other PTMs on USP13 could be influenced by Thr122 phosphorylation. Various combinations of PTMs on USP13 may result in diverse effects by altering its cellular localization, deubiquitinase enzymatic activity, or substrate specificity of USP13 in a different context of cancers. Further studies will determine whether CK2-mediated Thr122 phosphorylation affects the deubiquitinase activity of USP13, USP13-interactome network, or other PTMs on USP13. 

The USP13 gene is frequently amplified in patients with HGSOC [12]. Our previous study demonstrated that the depletion of USP13 selectively killed the USP13-highly expressing ovarian cancer cells and suppressed ovarian tumor growth in vivo, suggesting an oncogenic addictive phenotype of USP13-amplified ovarian cancer cells [12]. In the genetically engineered mouse model, USP13 promoted the development of highly aggressive epithelial serous ovarian tumors with rapid peritoneal metastasis and hemorrhagic ascites formation, which led to decreased survival [14]. These findings have suggested USP13 as a promising therapeutic target for ovarian cancer. CK2 is constitutively active and often overexpressed in several cancer types, including ovarian cancer [35,38,39]. The activity, expression, and cellular localization of CK2 components could further impact USP13 phosphorylation in response to intrinsic or extrinsic cellular stresses in the tumor microenvironment. In conclusion, we identified CK2-mediated phosphorylation of USP13 in ovarian cancer. Inhibition of the phosphorylation of USP13 downregulated USP13 protein stability and reduced the proliferation of ovarian cancer cells, which may lead to novel therapeutics targeting USP13 in USP13-amplified cancers.

## 4. Materials and Methods

### 4.1. Cell Culture and Drug Treatment

The human ovarian cancer cell lines HeyA8 and COV318 were obtained from the MD Anderson Characterized Cell Line Core Facility, which supplies authenticated cell lines. HEK293T cells were obtained from the American Type Culture Collection and cultured under standard conditions specified by the manufacturer. Cells were maintained in a DMEM medium (Thermo Fisher, Waltham, MA, USA) supplemented with 10% fetal bovine serum (Sigma-Aldrich, St. Louis, MO, USA) and 1% penicillin/streptomycin (Thermo Fisher) at 37 °C with 5% CO_2_. All cell lines tested negative for mycoplasma using a Mycoplasma Detection kit (Lonza). Immortalized fallopian epithelial cell lines (SV40 TAg-immortalized FT33, hTERT+shp53+CDK4R24C-immortalized FT237) were cultured as previously described [40,41,42] and were generously provided by Dr. Ronny Drapkin (University of Pennsylvania, Philadelphia, PA, USA). CK2 inhibitors (CX-4945 and TBB) were purchased from Selleck Chemicals (Selleckchem, Houston, TX, USA). Cell viability was determined by counting live cell numbers or by the 3-(4,5-dimethylthiazol-2-yl)-2,5-diphenyltetrazolium bromide (MTT) assay (Sigma-Aldrich), and absorbance was measured by a plate reader at 570 nm. Data are represented as the mean ± standard deviation. Student’s *t*-tests were performed to compare cell viability.

### 4.2. Western Blot

Cell lysates were extracted with 1% NP-40 lysis buffer containing a protease and phosphatase inhibitors cocktail (#87785, Thermo Fisher). Cell lysates were kept on ice for 30 min and centrifuged at 13,200 rpm for 10 min at 4 °C. Protein concentrations were measured by the Bio-Rad protein assay (#5000006, Bio-Rad, Hercules, CA, USA). A 4X Laemmli sample buffer (#1610747, Bio-Rad) containing 2-mercaptoethanol (#1610710, Bio-Rad) was added to the cell lysates and boiled at 95 °C for 10 min. Protein samples were separated on 4%-15% SDS-PAGE gels and transferred to polyvinylidene difluoride (PVDF) membranes (#1610710, Bio-Rad). The membranes were blocked with 5% BSA, or 5% skim milk in TBS-T (0.1% Tween-20) for 1 h at room temperature and incubated overnight at 4 °C with primary antibodies: Phospho-Threonine 122-USP13 (1:1000), USP13 (#sc-514416, 1:1000, Santa Cruz Biotechnology, Dallas, TX, USA), FLAG (#F1804, 1:1000, Sigma-Aldrich), β-actin (#sc-47778, 1:1000, Santa Cruz Biotechnology). The next day, the membrane was probed for 1 h at room temperature with an appropriate secondary antibody conjugated to horseradish peroxidase. Clarity Western ECL Substrate (170-5061, Bio-Rad) was applied for membrane development, and images were analyzed using the ChemiDoc MP system (Bio-Rad).

### 4.3. Immunoprecipitation

HeyA8 cells were transiently transfected with FLAG-tagged USP13 vector using Fugene HD reagent following the manufactural instruction (Promega, Madison, WI). Cells were lysed on ice for 30 min in IP buffer (1% NP-40, 50 mM Tris-HCl, 500 mM NaCl, 5 mM EDTA) containing a protease and phosphatase inhibitor cocktail. Cell lysates (1 mg) were incubated overnight with 1 µg of anti-FLAG M2 antibody (#F1804, Sigma-Aldrich) or normal IgG (12-371, Sigma-Aldrich) at 4 °C with rotary agitation. Protein A-Sepharose beads (Thermo Fisher) were added to the lysates and incubated for 4 h. Beads were washed three times with IP buffer and boiled for 10 min in 3% SDS sample buffer. Interaction between USP13 and CK2 was verified with western blotting.

### 4.4. Mass-Spectrometry Analysis and Database Search

Cells were lysed on ice for 30 min in IP buffer (1% NP-40, 50 mM Tris-HCl, 500 mM NaCl, 5 mM EDTA) containing a protease and phosphatase inhibitor cocktail. Cell lysates (1 mg) were incubated overnight with 3 µg of USP13 antibody (A302-762A, Bethyl Laboratories) or normal IgG (#2729, Cell Signaling Technologies) at 4 °C with rotary agitation. Protein A-Sepharose beads (Thermo Fisher) were added to the lysates and incubated for 4 h. Beads were washed three times with IP buffer and boiled for 10 min in 3% SDS sample buffer. NanoUPLC-MS/MS further analyzed immunoprecipitants after SDS-PAGE and Coomassie brilliant staining (BP36201, Thermo Fisher). The band was cut out and excised into cubes with a razor blade. Gel pieces were destained and digested with sequencing-grade trypsin. The tryptic peptide mixtures were analyzed by mass spectrometric analysis using NanoUPLC-MS/MS (Waters, Milford, MA) as previously described [43]. Data files were submitted for simultaneous searches using Protein Pilot version 5.0 software (Sciex) utilizing the Paragon and Progroup algorithms and the integrated false discovery rate (FDR) analysis function. MS/MS data were searched against the NCBI Homo Sapiens of the reviewed Uniprot-Sprot database. A customized USP13 protein database was also constructed in-house for database searching. The detected protein threshold in the software was set to the value corresponding to 1% FDR. Peptides were defined as redundant if they had identical cleavage site(s), amino acid sequence, and modification.

### 4.5. Generation of Anti-Phospho-Thr122 USP13 Antibody

The rabbit polyclonal antibody production was performed by Genscript USA, Inc. (Piscataway, NJ). In brief, phosphorylated peptide (CRRNSKIFLDLD(pThr)DD) was conjugated with keyhole limpet hemocyanin (KLH). Two New Zealand rabbits were immunized with conjugated peptide three times. After the third immunization, the phospho-specific antibody was purified by affinity purification (Genscript). The antibody’s immune reactivity was confirmed using indirect ELISA, with USP13 and pUSP13(T122) peptide used as coating antigens and a goat anti-rabbit IgG Fc monoclonal secondary antibody. Antigen-specificity was also verified by immunoblotting.

### 4.6. Protein Purification and CK2 Kinase Assay

The recombinant GST and GST-tagged USP13 were expressed in BL21 (DE3) E. coli cells. The bacterial cultures were grown at 37 °C until OD60 nm reached 0.6–0.8, and GST protein was then induced overnight with 1 mM Isopropyl β- d-1-thiogalactopyranoside (IPTG) at 30 °C. The cells were harvested in PBS (Thermo Fisher) containing protease inhibitors and lysed by sonication. Then the supernatant from the bacterial lysate was subjected to pull-down with on glutathione-Sepharose beads. After washing, the quantity of bead-bound GST or GST-tagged USP13 proteins was confirmed in SDS-gel running, followed by Coomassie brilliant blue staining. Active CK2 complex (P6010S), kinase buffer (B6022), and ATP (P0756S) were purchased from NEB (New England Biolabs, Ipswich, MA). The same amount of recombinant GST and GST-USP13 proteins was added into 1X kinase reaction buffer, including 200 μM of ATP and 1 μL of CK2. Kinase reaction was performed for 1 h at 30 °C with continuous shaking. After 1 h, the reactions were put on ice, supplemented with 5 μL 4× Laemmli sample buffer and 2 μL β-ME, and then boiled (5 min, 95 °C). Equal aliquots of all samples were separated by SDS-PAGE and analyzed by western blotting.

## 5. Conclusions

Phosphorylation of USP13 at Thr122 by CK2 increases the stability of USP13, which in turn promotes ovarian cancer proliferation. These results suggest that inhibition of Thr122 phosphorylation may be a therapeutic strategy for targeting USP13 in ovarian cancer.

## Data Availability

The data presented in this study is available in this article.
